# Hygiene Hypothesis Indicators and Prevalence of Antinuclear Antibodies in US Adolescents

**DOI:** 10.3389/fimmu.2022.789379

**Published:** 2022-01-28

**Authors:** Helen C. S. Meier, Dale P. Sandler, Jesse Wilkerson, Frederick W. Miller, Gregg E. Dinse, Christine G. Parks

**Affiliations:** ^1^ Population, Neurodevelopment and Genetics Program, Survey Research Center, Institute for Social Research, University of Michigan, Ann Arbor, MI, United States; ^2^ Epidemiology Branch, National Institute of Environmental Health Sciences (NIEHS), Durham, NC, United States; ^3^ Public Health & Scientific Research, Social and Scientific Systems, Durham, NC, United States; ^4^ Environmental Autoimmunity Group, National Institute of Environmental Health Sciences (NIEHS), Durham, NC, United States

**Keywords:** antinuclear antibodies (ANA), hygiene hypothesis, adolescents, asthma, allergy, autoimmunity

## Abstract

Autoimmunity prevalence, as measured by antinuclear antibodies (ANA), is increasing in U.S. adolescents. Improved hygiene and cleaner environments in childhood may reduce exposure to infections and other immune challenges, resulting in improper immune responses to later-life exposures. We examined associations of hygiene hypothesis indicators, including asthma, allergies, and antibodies to infectious agents, with ANA prevalence, measured by HEp-2 immunofluorescence, in adolescents (aged 12-19 years) over a 25-year time span in the National Health and Nutrition Examination Survey (NHANES) (N=2,709), adjusting for age, sex, race/ethnicity, body mass index, education and survey cycle, overall and within individual time periods, using logistic regression. Prevalence of ANA in adolescents increased from 5.0% in 1988-1991 to 12.8% in 2011-2012. ANA were positively associated with diagnosis of asthma in early childhood (OR: 2.07, CI: 1.09–3.99) and the effect estimate for current hay fever was elevated but not statistically significant (OR: 1.55, CI: 0.85–2.84). Fewer than 2% of those with ANA in 1988-1991 had been diagnosed with asthma, compared with 18% in 1999-2000, and 27% in 2003-2004 and 2011-2012. ANA trended negatively with *Helicobacter pylori* antibodies (OR: 0.49, CI: 0.24–0.99). ANA may be useful as an additional indicator of inadequate immune education in adolescence, a critical period of growth and development.

## 1 Introduction

The prevalence of autoimmunity, characterized as the immune system responding to non-threatening self-antigens, was recently shown in a nationally representative sample of U.S. adolescents to have increased substantially over the last 25 years (1). Drivers of this trend remain unknown (1). Developing immune systems in children and adolescents require education through immune challenges to function properly later in life (2). The hygiene hypothesis posits that improved hygiene and clean environments in childhood lead to declining exposure to infections and other historically common immune challenges and to improper immune responses to non-threatening exposures, such as allergens later in life (3–6). Indeed, asthma and allergy are commonly used as indicators for inadequate immune challenge in childhood (7) and asthma prevalence in adolescents has also steadily increased over time while the prevalence of common infection indicators has decreased (8, 9).

**Figure 1:**
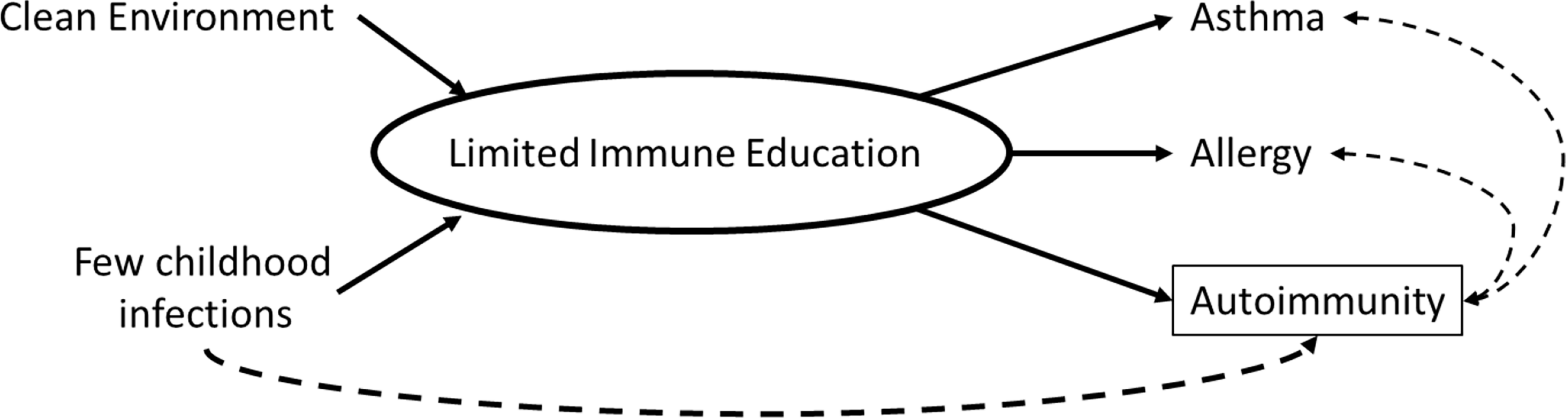
Conceptual diagram of the relationship between hygiene hypothesis indicators and autoimmunity.

Immune mechanisms underlying the hygiene hypothesis are not fully-established but include the possibility that reduced microbial exposures in early life may prevent the activation of regulatory T-cells, thereby enabling both the development of allergic diseases and activation of autoimmune pathways (4). To elucidate whether autoimmunity, measured by antinuclear antibodies (ANA), may be an additional indicator of inadequate immune system education in childhood and adolescence, we explored age and birth cohort associations of common hygiene hypothesis indicators, including asthma, allergy, and persistent infections, with the presence of ANA in a representative sample of U.S. adolescents from the National Health and Nutrition Examination Survey (NHANES) (**Figure 1**).

We hypothesized that an increase in allergy and asthma prevalence seen over 25 years in the U.S. would be correlated with the increase in ANA prevalence observed in adolescents. Further, we hypothesized that lower prevalence of common types of infections, would be inversely associated with ANA prevalence.

## 2 Materials and Methods

The study population was all adolescents, aged 12-19 years, from four cross-sectional NHANES cycles for which ANA data were available (overall sample, N=2,709): 1988-1991 (N=676), 1999-2000 (N=609), 2003-2004 (N=581) and 2011-2012 (N=843). The 2001-2002 cycle was not included as no individuals aged 12-19 were tested for ANA. NHANES is a representative survey of the non-institutionalized U.S. population. Provided subsample specific weights were used to adjust for nonresponse and the probability of selection into each ANA subsample (10). The NHANES protocol was approved by the National Center for Health Statistics (NCHS) Ethics Review Board of the Centers for Disease Control and Prevention (CDC). All participants or guardians gave written informed consent. Laboratory assays were conducted previously as part of standardized protocols available at: https://wwwn.cdc.gov/nchs/nhanes/Search/DataPage.aspx?Component=Laboratory.

### 2.1 Outcome

ANA were measured across four time periods as described previously (1) with indirect immunofluorescence at a 1:80 dilution using the NOVA Lite HEp-2 ANA slide with DAPI kit (INOVA Diagnostics, San Diego, CA) with a highly specific fluorescein isothiocyanate (FITC)-conjugated secondary antibody (goat anti-human IgG). Images were captured *via* the NOVA View automated fluorescence microscope system (INOVA Diagnostics) and stored digitally. Staining intensities were graded from 0 to 4 relative to a standard reference gallery, with non-zero values (e.g., values 1-4) considered indicative of ANA positivity (1). All samples from all years were assayed at the same time, using the same methods in a single laboratory. Readings were made independently by at least two experienced evaluators (blinded to sample characteristics and time period), who agreed on >95% of the intensities and patterns; differences were resolved by consensus or adjudicated by a third blinded rater. Repeat testing of random samples showed >98% concordance.

### 2.2 Exposures

Except where specified, data on exposures were available across all four cycles. Serology methods varied for Toxoplasma and Herpes assays by cycle as described below.

#### 2.2.1 Asthma

Participants were asked if a “doctor or other health professional ever told you that you have asthma.” Affirmative responders were then asked, “How old were you when you were first told you had asthma?” Responses were categorized as, asthma diagnosed before age 6, asthma diagnosed at age 6 or later, or no asthma diagnosis (referent). Current asthma was ascertained by asking, “Do you still have asthma?”

#### 2.2.2 Allergy

Participants were asked, “During the past 12 months, have you had hay fever?” Responses were categorized as “yes” or “no”. Atopic asthma was derived by combining self-reported current asthma and hay fever status into a four-level categorical variable: no atopy (referent), hay fever only, current asthma only, and both hay fever and current asthma.

#### 2.2.3 Infections

##### 2.2.3.1 *Helicobacter pylori* (*H. pylori*) Seropositivity – 1988-1991 and 1999-2000 Only

*H. pylori* antibody testing was previously performed at the University of Washington on surplus sera. *H. pylori*-specific IgG was measured using Wampole Laboratories (Cranbury, NJ) *H. Pylori* IgG Enzyme-Linked Immunosorbent Assay (ELISA). Standard ELISA cut-offs were used to categorize participants into seropositive (optical density (OD) value >1.1) or seronegative (OD value <0.9) to *H. pylori*. Equivocal values (0.9-1.1) were categorized as seronegative to produce conservative estimates of serostatus.

##### 2.2.3.2 *Toxoplasma gondii* (*T. gondii*) Seropositivity

1988-1991: The presence and quantity of antibodies to *T. gondii* were determined by comparing the OD of the test sample to a standard curve constructed using optical density readings from positive control sera obtained from a kit; these readings were calibrated to WHO Toxo 60 serum and read as International Units (IU/mL). Test samples with results ≥7 IU/mL were considered to be positive, indicating infection at some undetermined time.

1999-2000 and 2003-2004: IgG to *T. gondii* was measured using a solid-phase enzyme immunoassay. Using OD readings from positive controls, a standard curve was constructed and calibrated to WHO Toxo 60 serum. Samples with OD ≥10 IU/mL were determined to be positive for *T. gondii* infection.

2011-2012: IgG to *T. gondii* was measured using an enzyme immunoassay. Samples with ≥33 IU/mL were coded as positive for *T. gondii* infection.

##### 2.2.3.3 Herpes Simplex Virus-1 (HSV-1) Seropositivity

All time periods (1988-1991, 1999-2000, 2003-2004 and 2011-2012): Sera from participants aged 14-18 were tested using solid-phase enzymatic immunodot assay specific for HSV-1 glycoprotein at Emory University, Atlanta, GA (11). Serum reactive to an immunodot containing gG-1 indicated previous and probable latent HSV-1 infection and was classified as seropositive.

### 2.3 Covariates

Covariates, chosen based on *a priori* knowledge of their relationships with the exposures and ANA positivity, included age (years), self-reported sex (male or female), race/ethnicity, education of reference parent, and body mass index (BMI). Race/ethnicity was categorized into Non-Hispanic White, Non-Hispanic Black, or other. Education of reference parent was categorized as less than high school, high school, or more than high school. BMI categories were calculated using CDC growth curve percentiles and classified as underweight/normal (<85^th^ percentile), overweight (85^th^ to <95^th^ percentile), or obese (≥95^th^ percentile). Tobacco smoke exposure, country of birth, and household size were included in descriptive characterization of ANA status. Tobacco smoke exposure was classified based on serum cotinine levels as smoker (>15ng/mL), secondhand exposure (limit of detection-15ng/mL), and no exposure (below the limit of detection). Country of birth was dichotomized as U.S. or other. Household size was categorized as 1-3 individuals, 4-5 individuals, or 6+ individuals.

### 2.4 Analyses

Prevalence of ANA was estimated for each time period both overall and by participant characteristics, including demographics, health status, and infection seropositivity. Unadjusted associations between ANA positivity and each characteristic were assessed *via* Rao-Scott χ^2^-tests for categorical characteristics and F-tests for continuous characteristics. Prevalence odds ratios (ORs) and 95% confidence intervals (CIs) from logistic regression were used to assess associations between ANA and each characteristic within and across all time periods, adjusted for age, sex, race/ethnicity, BMI, and education of reference parent. Sensitivity analyses additionally adjusted for tobacco smoke exposure, country of birth and household size.

Assessment of ANA trend over time was based on two adjusted logistic regression models. The first model added a categorical covariate for time period and estimated the ORs and 95% CIs for ANA positivity in each period, relative to the first. The second model instead added a quantitative covariate for the number of years between period midpoints, relative to the first (0, 10, 14, and 22 years), and produced a P-value from a χ^2^-test to assess an ANA positivity time trend. Both time trend logistic regression models were each adjusted for age, sex, race/ethnicity, BMI, and education of reference parent. All analyses were performed using SAS software survey procedures (version 9.4; SAS Institute, Cary, NC). Sampling weights and design variables were incorporated in all analyses to obtain unbiased national prevalence and variance estimates.

## 3 Results

### 3.1 Cross-Sectional Analyses by Time Period

**Figure 2:**
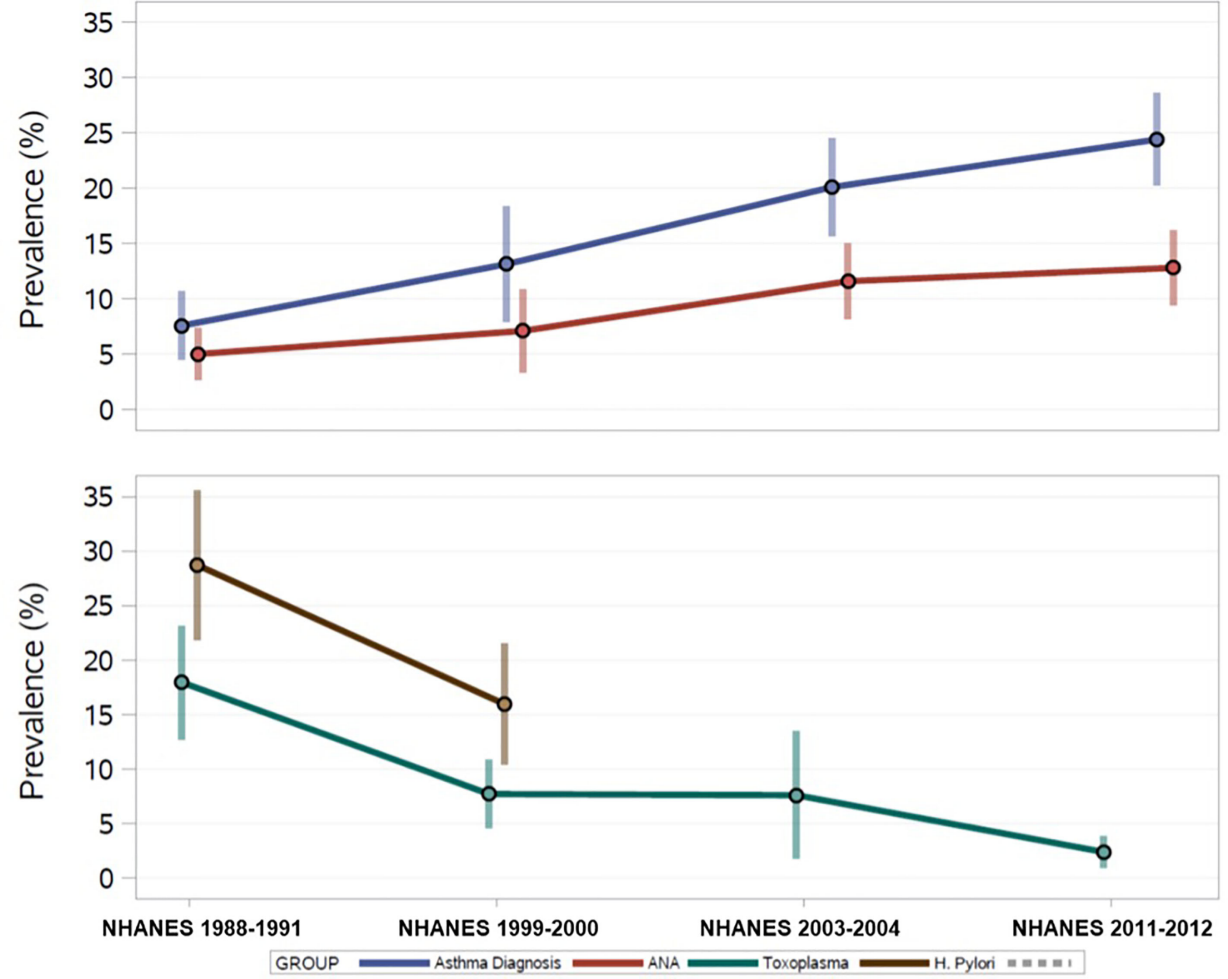
U.S. Prevalence Estimates of ANA, Asthma, and Persistent Infections over 25 years from the National Health and Nutrition Examination Survey, 1988-2012.

The percent of ANA positive adolescents increased over time from 5.0% in 1988-1991 to 12.8% in 2011-2012 (**Figure 2**). The prevalence of asthma also increased over that time span, while the prevalence of infection indicators, such as antibodies to *H.pylori, T. gondii*, and HSV-1, has steadily decreased over time.

The distributions of participant characteristics by ANA status for each time period are depicted in **Table 1**. Within most time periods, ANA positive individuals were more likely to be female, but no other associations between ANA and demographic characteristics emerged consistently across time periods. ANA were not patterned by age, race/ethnicity, education, or smoking. Several characteristics exhibited associations with ANA in specific time periods. In 2003-2004, ANA positive individuals were less likely to be obese than ANA negatives and ANA positivity varied by household size. In 1999-2000, ANA positives were more likely to be born in the U.S. than ANA negative individuals.

**Table 1:** Participant Characteristic Counts and Weighted Proportions by Time Period and ANA Status in U.S. Adolescents.

Characteristic	ANA Status NHANES 1988-1991			ANA Status NHANES 1999-2000			ANA Status NHANES 2003-2004			ANA Status NHANES 2011-2012			
	Negative	Positive		Negative	Positive		Negative	Positive		Negative	Positive		Overall
	(n = 631, 95.0%)	(n = 45, 5.0%)		(n = 555, 92.9%)	(n = 54, 7.1%)		(n = 521, 88.4%)	(n = 60, 11.6%)		(n=741, 87.2%)	(n = 102, 12.8%)		(N = 2709)
	n (%)	n (%)	P-value	n (%)	n (%)	P-value	n (%)	n (%)	P-value	n (%)	n (%)	P-value	n (%)
**Demographics**													
Sex													
Female	325 (48.1)	35 (67.8)	0.086	257 (45.2)	36 (66.1)	0.034	239 (48.1)	46 (73.5)	0.015	349 (47.8)	60 (55.9)	0.284	1347 (49.0)
Male	306 (51.9)	10 (32.2)		298 (54.8)	18 (33.9)		282 (51.9)	14 (26.5)		392 (52.2)	42 (44.1)		1362 (51.0)
Age (Years)													
12-13	161 (24.1)	7 (27.0)	0.437	153 (23.0)	18 (22.9)	0.896	133 (27.8)	9 (13.2)	0.193	186 (24.9)	28 (27.5)	0.719	695 (24.7)
14-15	126 (18.1)	17 (24.8)		123 (24.1)	12 (27.7)		122 (23.8)	15 (26.6)		186 (26.0)	19 (26.8)		620 (23.6)
16-17	175 (29.0)	13 (36.9)		145 (25.1)	8 (27.6)		136 (25.1)	19 (39.0)		178 (24.1)	26 (17.7)		700 (26.0)
18-19	169 (28.8)	8 (11.2)		134 (27.8)	16 (21.8)		130 (23.2)	17 (21.2)		191 (25.0)	29 (28.1)		694 (25.7)
Race/Ethnicity													
White	202 (67.9)	9 (57.4)	0.298	116 (57.2)	11 (54.2)	0.239	136 (64.8)	16 (67.4)	0.871	167 (54.4)	27 (63.2)	0.102	684 (61.1)
Black	177 (15.5)	9 (14.2)		138 (12.8)	18 (22.5)		192 (15.7)	20 (12.5)		219 (15.3)	31 (15.1)		804 (14.8)
Other	252 (16.6)	27 (28.3)		301 (30.0)	25 (23.3)		193 (19.5)	24 (20.1)		355 (30.3)	44 (21.7)		1221 (24.1)
Body Mass Index													
Underweight/Normal	470 (78.0)	34 (76.6)	0.963	330 (67.7)	35 (81.2)	0.062	296 (56.1)	46 (83.8)	0.001	449 (63.1)	69 (69.4)	0.405	1729 (66.3)
Overweight	72 (10.1)	7 (12.2)		91 (14.9)	6 (6.2)		96 (19.5)	9 (6.0)		113 (15.5)	17 (16.7)		411 (15.0)
Obese	89 (11.9)	4 (11.1)		130 (17.4)	13 (12.6)		128 (24.3)	5 (10.2)		164 (21.3)	13 (13.9)		546 (18.6)
Missing	0	0		4	0		1	0		15	3		23
Education of Reference Parent													
Less than High School	292 (28.8)	23 (31.8)	0.952	234 (27.3)	24 (25.6)	0.979	179 (20.5)	19 (19.8)	0.381	198 (25.1)	22 (13.3)	0.052	991 (24.6)
High School Grad/GED or Equivalent	178 (32.7)	10 (31.3)		134 (26.8)	11 (26.3)		116 (27.1)	12 (17.7)		166 (22.3)	30 (20.2)		657 (26.6)
Some College or Above	160 (38.5)	11 (36.9)		158 (46.0)	16 (48.1)		200 (52.4)	27 (62.4)		346 (52.7)	49 (66.5)		967 (48.8)
Missing	1	1		29	3		26	2		31	1		94
Smoking (Cotinine)													
Smoking (≥15ng/mL)	96 (21.7)	4 (3.7)	0.017	79 (21.7)	7 (7.2)	0.170	68 (16.3)	2 (5.6)	0.321	64 (8.5)	7 (6.9)	0.888	327 (16.2)
Secondhand (LOD-15ng/mL)	466 (69.8)	35 (88.1)		290 (55.4)	26 (61.9)		384 (71.2)	45 (80.3)		461 (60.9)	71 (61.7)		1778 (64.8)
Not detected (BLOD)	50 (8.5)	5 (8.2)		178 (22.9)	21 (30.8)		69 (12.5)	13 (14.1)		216 (30.6)	24 (31.4)		576 (18.9)
Missing	19	1		8	0		0	0		0	0		28
Household Size													
1-3	136 (26.7)	7 (29.9)	0.171	120 (31.7)	16 (35.8)	0.459	130 (29.4)	12 (7.6)	<0.001	185 (25.6)	21 (26.7)	0.251	627 (28.1)
4-5	304 (52.4)	20 (36.6)		241 (47.6)	23 (53.0)		246 (52.0)	35 (83.4)		382 (53.1)	58 (61.3)		1309 (52.4)
6+	191 (20.9)	18 (33.4)		194 (20.7)	15 (11.2)		145 (18.6)	13 (9.0)		174 (21.3)	23 (12.0)		773 (19.5)
Birth Country													
United States	523 (89.0)	34 (93.2)	0.361	451 (88.8)	48 (97.0)	0.008	453 (91.0)	52 (87.3)	0.565	617 (89.4)	94 (93.5)	0.251	2273 (89.8)
Other	103 (11.0)	11 (6.8)		104 (11.2)	6 (3.0)		68 (9.0)	8 (12.7)		124 (10.6)	8 (6.5)		432 (10.2)
Missing	4	0		0	0		0	0		0	0		4
**Infection Seropositivity**													
*Helicobacter pylori*	212 (29.0)	15 (23.6)	0.588	150 (16.6)	10 (7.9)	0.111							387 (20.5)
*Toxoplasma gondii*	112 (18.1)	5 (14.7)	0.669	57 (7.0)	5 (16.9)	0.127	29 (7.1)	4 (11.2)	0.427	21 (2.3)	5 (3.2)	0.524	238 (8.4)
Herpes Simplex Virus- 1^a^	251 (45.1)	20 (24.3)	0.057	216 (40.6)	21 (44.5)	0.686	185 (38.6)	21 (31.9)	0.394	222 (35.1)	33 (32.0)	0.584	969 (39.1)
**Health Status**													
Ever asthma diagnosis	52 (7.9)	1 (1.8)	0.076	71 (12.7)	6 (18.1)	0.531	86 (19.2)	17 (27.0)	0.322	156 (24.1)	24 (26.7)	0.599	413 (16.8)
Current asthma	38 (5.7)	1 (1.8)	0.191	46 (8.2)	3 (7.4)	0.899	56 (10.4)	12 (17.0)	0.379	90 (14.7)	13 (13.1)	0.801	259 (10.0)
Asthma diagnosis age (n=400)													
< 6 years old	26 (59.3)	0 (0.0)	NA	30 (39.9)	3 (39.8)	0.998	46 (54.2)	10 (77.4)	0.084	85 (51.3)	10 (74.0)	0.019	210 (52.6)
6 years or older	20 (40.7)	1 (100.)		41 (60.1)	3 (60.2)		39 (45.8)	6 (22.6)		68 (48.7)	12 (26.0)		190 (47.4)
Current hay fever	49 (10.3)	3 (4.2)	0.157	60 (10.1)	9 (13.0)	0.545	44 (9.9)	6 (18.1)	0.158	83 (13.0)	17 (20.9)	0.304	271 (11.2)
Atopic Asthma													
Current asthma and hay fever	8 (1.5)	0 (0.0)	NA	13 (2.5)	1 (1.1)	0.778	11 (2.3)	2 (4.3)	0.556	21 (4.6)	2 (5.3)	0.634	58 (2.8)
Current hay fever only	41 (8.7)	3 (4.2)		46 (7.5)	8 (11.9)		33 (7.6)	4 (13.8)		62 (8.4)	15 (15.6)		212 (8.4)
Current asthma only	30 (4.2)	1 (1.8)		33 (5.8)	2 (6.4)		45 (8.2)	10 (12.7)		68 (9.7)	11 (7.7)		200 (7.2)
No atopy	551 (85.5)	41 (94.0)		459 (84.2)	42 (80.6)		431 (81.9)	44 (69.2)		584 (77.3)	74 (71.3)		2226 (81.6)

a Ages 14-19 Only.

Overall, 17% of the sample reported an asthma diagnosis, with the prevalence steadily increasing over time (**Figure 2**). **Table 2** shows the adjusted prevalence ORs and 95% CIs for assessing ANA associations with asthma and allergy by time period and across all time periods combined. “Ever” asthma diagnosis was not associated with ANA positivity in any individual time period, however, OR estimates were elevated in 1999-2000 and 2003-2004. Overall, individuals with an asthma diagnosis had 1.46 times the adjusted odds of ANA positivity as individuals who had never been diagnosed with asthma (95% CI: 0.84, 2.54), though this effect estimate was not statistically significant at the 0.05 level.

**Table 2:** Prevalence Odds Ratios and 95% Confidence Intervals Assessing ANA Association with Hygiene Hypothesis Indicators by NHANES Time Period Adjusted for Age, Sex, Race/ethnicity, BMI and Education of Reference Parent.

	NHANES 1988-1991		NHANES 1999-2000		NHANES 2003-2004		NHANES 2011-2012		Overall		Trend^a^ OR (95% CI)	Trend p-value
	OR (95% CI)	p-value	OR (95% CI)	p-value	OR (95% CI)	p-value	OR (95% CI)	p-value	OR (95% CI)	p-value		
***Asthma and allergy***												
Asthma Diagnosis (Ever *vs*. No)	0.22 (0.03-1.42)	0.111	1.80 (0.50-6.54)	0.371	2.12 (0.85-5.30)	0.106	0.97 (0.60-1.57)	0.892	1.46 (0.84-2.54)	0.185	1.05 (1.03-1.08)	<0.001
Asthma Diagnosis (Age of Diagnosis)^b^												
Age < 6									2.07 (1.08-3.99)	0.029	1.05 (1.03-1.08)	<0.001
Age 6+									0.87 (0.42-1.83)	0.715		
No asthma									1.00	Ref		
Current Asthma	0.31 (0.05-2.08)	0.229	1.00 (0.15-6.67)	0.996	3.15 (1.00-9.91)	0.050	0.80 (0.28-2.30)	0.681	1.29 (0.58-2.87)	0.532	1.06 (1.03-1.08)	<0.001
Hay Fever	0.43 (0.09-1.95)	0.273	1.32 (0.53-3.30)	0.549	2.57 (0.89-7.47)	0.082	1.32 (0.47-3.66)	0.598	1.55 (0.85-2.84)	0.153	1.06 (1.03-1.08)	<0.001
Atopic asthma												
Current asthma and hay fever	———–	——	0.46 (0.05-3.83)	0.469	5.60 (1.05-29.83)	0.044	0.97 (0.12-8.02)	0.979	1.28 (0.34-4.85)	0.717	1.06 (1.03-1.08)	<0.001
Hay fever only	0.48 (0.10-2.21)	0.347	1.65 (0.54-5.03)	0.378	2.47 (0.48-12.68)	0.279	1.47 (0.52-4.15)	0.469	1.73 (0.77-3.86)	0.183		
Current asthma only	0.39 (0.05-2.98)	0.361	1.35 (0.14-13.02)	0.797	3.03 (0.84-10.96)	0.091	0.81 (0.35-1.88)	0.618	1.42 (0.61-3.34)	0.417		
No atopy	1.00	Ref	1.00	Ref	1.00	Ref	1.00	Ref	1.00	Ref		
***Infection Seropositivity***												
* Helicobacter pylori*	0.67 (0.19-2.33)	0.525	0.33 (0.14-0.78)	0.011					0.49 (0.24-0.99)	0.047		
* Toxoplasma gondii*	0.80 (0.24-2.73)	0.725	2.27 (0.59-8.73)	0.234	3.06 (0.77-12.14)	0.113	2.41 (0.86-6.76)	0.095	1.86 (0.88-3.93)	0.103	1.06 (1.04-1.09)	<0.001
Herpes Simplex-1	0.19 (0.07-0.52)	0.001	0.95 (0.40-2.22)	0.897	0.83 (0.37-1.86)	0.648	0.77 (0.52-1.15)	0.197	0.74 (0.48-1.13)	0.164	1.06 (1.02-1.09)	0.001

a Odds of ANA positivity from NHANES 1988-1991 through NHANES 2011-2012.

b Among Asthmatics.

Of those reporting ever having asthma and a diagnosis age (n=400), 53% were diagnosed before the age of 6 years and the proportion of ANA positive individuals who were diagnosed with asthma before age 6 increased from 40% in 1999-2000 to 74% in 2011-2012 (**Table 1**). Individuals with an asthma diagnosis before the age of 6 had 2.07 times the adjusted odds of ANA positivity as those without asthma (95% CI: 1.08, 3.99) (**Table 2**). This association was robust to additional adjustment for smoking, country of birth, and household size. No association was observed between asthma diagnosed at age 6 or later and ANA positivity. Approximately 10% of participants reported current asthma in the full sample, and no consistent associations with ANA positivity were observed.

Self-report of hay fever, representing allergy, was present in 11% of adolescents overall. In all time periods except 1988-1991, a higher proportion of ANA positive individuals reported hay fever than ANA negative individuals, reaching 21% in 2011-2012 (**Table 1**). Overall, individuals with hay fever had 1.55 times the adjusted odds of ANA positivity as those without hay fever, although the 95% confidence interval contains the null value (95% CI: 0.85, 2.84, **Table 2**). The largest period-specific OR was 2.57 (95% CI: 0.89, 7.74) in 2003-2004.

Few adolescents in this study reported both asthma and hay fever (i.e., “atopic asthma” 2.8%), and most experienced neither (81.6%) (**Table 1**). There were no observed differences by sex (**Supplemental Table 1**). In 2003-2004, those with atopic asthma had 5.6 times the adjusted odds of ANA positivity as adolescents with neither asthma nor hay fever (95% CI: 1.05, 29.83) (**Table 2**). This association was robust to additional adjustment for country of birth, household size, and smoking.

*H. pylori* seroprevalence decreased from 1988-1991 to 1999-2000 from 34% to 26% among adolescents with ANA results. Among all adolescents assayed for *H. pylori*, regardless of ANA testing, seropositivity decreased from 28% to 16% (**Figure 2**). A greater proportion of male adolescent ANA positive individuals were *H. pylori* seropositive than females in 1988-1991, but this was not observed in 1999-2000. *H. pylori* in adolescents was inversely associated with ANA positivity (overall OR: 0.49, 95% CI: 0.24, 0.99), with the stronger association observed in 1999-2000 (OR: 0.33, 95% CI: 0.14, 0.78) (**Table 2**).

*T. gondii* seroprevalence decreased over time from 17% in 1988-1991 to 2.5% in 2011-2012 (**Figure 2**). Decreasing frequency of *T. gondii* by ANA status was observed with just under 15% of ANA positives also being *T. gondii* seropositive in 1988-1991 decreasing to about 3% in 2011-2012. Overall, adolescents who were seropositive for *T. gondii* had 1.86 higher adjusted odds of ANA positivity than seronegative adolescents (95% CI: 0.88, 3.93), and all time periods except 1988-1991 had elevated ORs (**Table 2**).

In adolescents aged 14-19, HSV-1 seroprevalence was 39% overall. No statistically significant difference in HSV-1 prevalence by ANA status was observed in any of the time periods, though seroprevalence among ANA positives increased from 24% in 1988-1991 to 45% in 1999-2000, declining to 32% in 2003-2004 and 2011-2012. HSV-1 seropositivity was inversely associated with ANA in 1988-1991 (OR: 0.19, 95% CI 0.07, 0.52) but this association was not statistically significant in other time periods.

### 3.2 Time Trend Analyses

The adjusted odds of ANA positivity increased by approximately 5% per year in U.S. adolescents from 1988-1991 through 2011-2012 (trend p-value <0.001; **Table 2**). This effect estimate was largely unchanged by additional adjustment for any of the exposures considered in this study, meaning they did not statistically explain the observed time trend. Since *H. pylori* seropositivity data were only available in 1988-1991 and 1999-2000, that infection was excluded from trend analysis.

**Figure 3:**
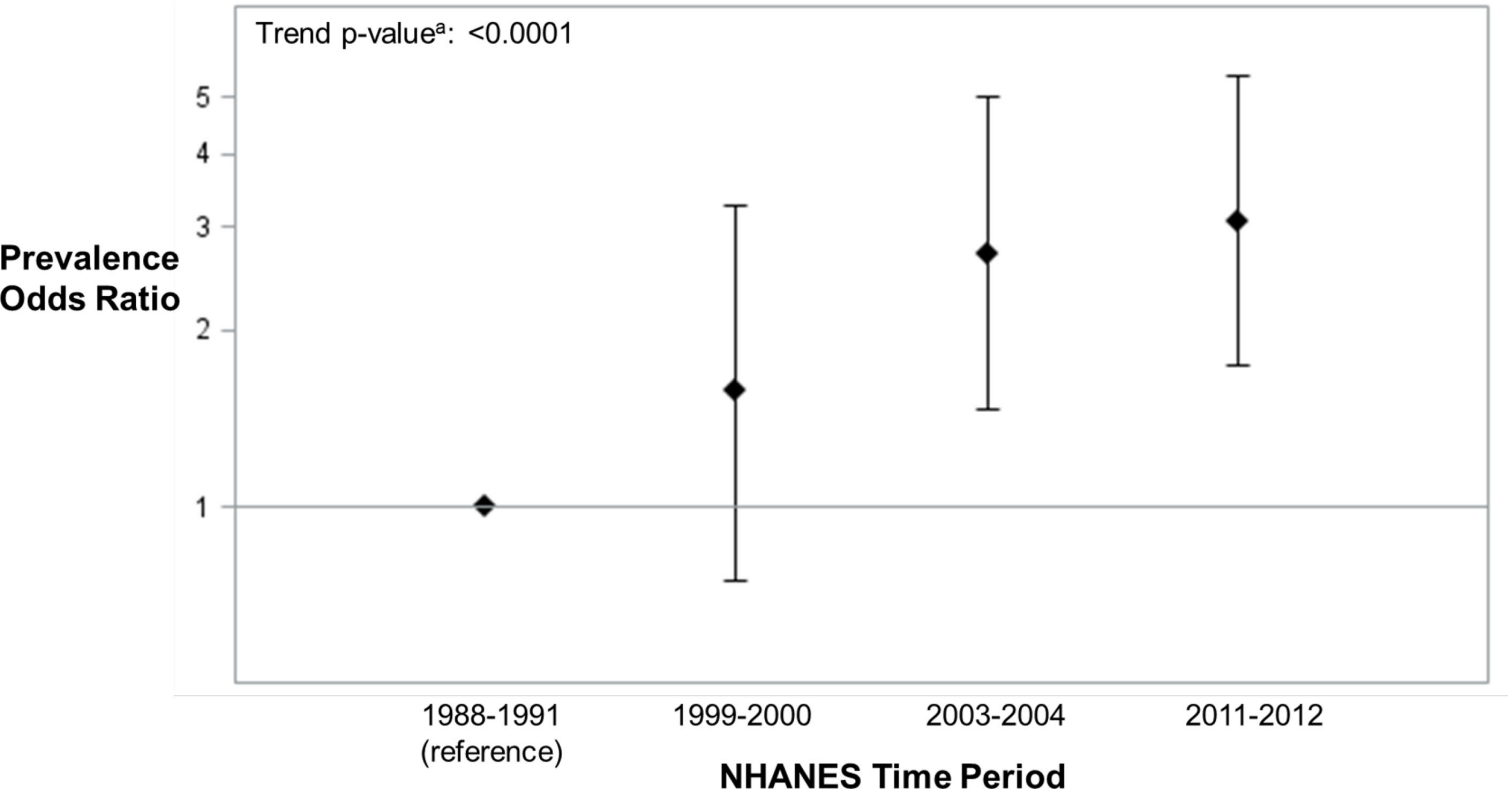
ANA Prevalence Odds Ratios (95% Confidence Intervals) in U.S. Adolescents by NHANES Time Period (n=2594) adjusted for age, sex, race/ethnicity, BMI and education of reference parent. ^a^The ANA time trend assessments were based on two logistic regression models that adjusted for the survey design variables (strata, clusters, and sampling weights) and categorical covariates. One model added a categorical covariate for time period and estimated the ANA prevalence odds ratio for each period, relative to the first. The other model added a quantitative covariate for the number of years between period midpoints, relative to the first, and produced a P-value from a χ^2^-test to assess an ANA time trend.

In assessing the relative risk of ANA positivity in each time period individually compared to 1988-1991 (referent), the adjusted odds of ANA positivity in adolescents over time increased from 1.57 (95% CI: 0.75, 3.28), to 2.71 (95% CI: 1.47, 5.00), and 3.07 (95% CI: 1.74, 5.42) in 1999-2000, 2003-2004, and 2011-2012, respectively (**Figure 3**), replicating previous analyses (1). Additional adjustment for country of birth, household size, and smoking did not meaningfully alter these results.

### 3.3 Additional Analyses

Age-adjusted prevalence ORs and 95% CIs for the association between potential model covariates and ANA positivity by time period and overall are reported in **Supplemental Table 2**. We also looked at ANA staining patterns, but no clear time trends were seen in the proportion of patterns (**Supplemental Table 3**). Compared to a NHANES sample including adolescents and adults (i.e. ages 12+) (1), in our study of adolescents only, periods 1 and 3 show a higher proportion had dense fine speckled and total mitotic staining, and we saw a lower proportion with cytoplasmic staining across all periods. In addition, we examined elevated ANA staining intensity (3 or 4+) by time period and found that the 2011-2012 time period had a higher proportion of adolescents with 3 or 4+ relative to the other time periods (**Supplemental Table 3**). However, due to a small sample size, this should be replicated in a larger sample.

## 4 Discussion

ANA prevalence has steadily increased in U.S. adolescents over a recent 25-year span (1). Adolescence is a time of immense change in physical growth and development. In particular, immune system development extends from the prenatal period through adolescence (12), and immune competence continually builds as neoantigens are encountered (13). Due to the pace of growth and hormonal shifts, late childhood and adolescence is a sensitive period where adolescents are particularly vulnerable to environmental exposures (13). The observed increase in ANA among adolescents, a 200% increase in the odds of ANA in 2011-12 compared to 1988-1991, is alarming and may represent temporal shifts in environmental exposures for this age range. We investigated whether the increase in adolescent ANA prevalence correlated with other factors associated with improved hygiene and cleaner environments in childhood, consistent with the hygiene hypothesis. Overall, our findings were mixed. Increases in ANA prevalence over time did not appear to be explained by asthma, allergy, or the infections that we studied.

However, we did observe a positive association between asthma diagnosis before 6 years and ANA positivity. It is possible that the airway inflammation (14) characteristic of this asthma phenotype and ANA may have a common underlying cause, including early life exposures. The co-occurrence of ANA and early-onset asthma may become more common in U.S. adolescents, though additional data from more proximal time periods is needed to monitor for a potential trend. Understanding the pathophysiological pathways that may link early-onset asthma and biomarkers of autoimmunity will be an important area of future research.

Though we did not observe consistent inverse relationships between the infections examined and ANA, as would be expected under the hygiene hypothesis, the data did suggest an inverse association between *H. pylori* and ANA positivity. *H. pylori* was previously shown to be protective for early-onset (less than 5 years) childhood asthma in NHANES (15). *H. pylori* reduces host inflammatory responses, even early in life, which in animal models have been shown to provide protective effects against allergic airway inflammation in first and second generation offspring (16–18). As with other persistent pathogens, the early life benefits of *H. pylori* infection may wane and undermine the mature adult immune system, resulting in detrimental outcomes (19–21). Indeed, a recent study of *H. pylori* in U.S. adults aged 20+ years found that *H. pylori* seropositivity was associated with higher odds of ANA positivity, suggesting that chronic inflammation resulting from *H. pylori* may impact immunological regulation (22). Additional research is needed to understand the relationship of persistent pathogens, such as *H. pylori*, and immune self-tolerance over the life course.

Elevated odds of ANA were observed for adolescents with atopic asthma in one time period (2003-2004), but these results were not replicated in other periods. Overall, atopic asthma was rare in adolescents (2.8%), so analyses were underpowered. Previous research on atopic asthma and autoimmune disease, in part motivated by the idea of antagonism between allergic and autoimmune responses, has shown mixed results spanning positive, negative, and no associations, with heterogenous definitions of autoimmune disease and atopy (23). Cross-regulatory T-helper Type 1 (TH1) versus Type 2 (TH2) immune responses have been hypothesized to lead to negative relationships or antagonism between allergic and autoimmune diseases (24, 25). According to the contemporary hygiene hypothesis, cleaner environments and less exposure to infections provide inadequate education of the immune system, resulting in higher likelihood of allergic and autoimmune disease. Thus, we would expect the occurrence of atopic asthma and autoimmunity to be correlated (26). Whether the co-occurrence is due to the same underlying dysregulation of immunological processes is an area of active research. Studies investigating atopic dermatitis in children have shown increased risk for autoimmune diseases (27). Studies have found positive associations between IgE autoantibodies and atopic dermatitis in children (26), and that children with atopic dermatitis develop ANA earlier than children without atopic dermatitis (27), suggesting potential for an underlying common cause including autoreactive T-cells and molecular mimicry (26). Further research on atopic asthma and autoimmunity is needed to understand common underlying mechanisms.

Our study has several limitations. We were limited to self-reported allergy, asthma and atopy information because NHANES did not link to participant medical records. Further, we do not have information on family history of autoimmune disorders because this was not asked in the NHANES surveys. Some of the conditions and infections examined had low prevalence, reducing power and precision of statistical analyses. Other infections examined in studies of the hygiene hypothesis, such as Hepatitis A (28), were not included due to vaccine licensure and varying vaccine updates during the study period. Previous studies have shown that ANA are more commonly detected during infections, and though autoimmunity may have a positive role during certain infections (29), in our study, ANA were not associated with self-reported infection (head or chest cold) in the last four weeks. The serological data in NHANES are cross-sectional so we cannot determine the temporal relationships of infections with ANA incidence. Longitudinal studies are warranted to better understand the age at onset and persistence of ANA, particularly in existing pediatric cohorts including biomarkers related to allergy and asthma phenotype (30). Detection of ANA by immunofluorescence identifies a wide range of autoantibodies and staining patterns. While we did not have data on disease-specific autoantibodies, these are uncommon in the general population (<10%) (31). The clinical interpretation of specific patterns is an important area of ongoing research. There is little known about ANA patterns in children and the natural history of ANA over time as they age into adulthood, though studies suggest varying factors, such as genetic variability and immune phenotype may be related to risk of developing autoimmune diseases (32). Investigation of specific cellular patterns with atopy is an important area for future research.

Despite these limitations, our study has strengths. We used nationally representative data spanning 25 years to understand increasing prevalence of ANA in U.S. adolescents. We contrasted ANA and serostatus to several infections whereas previous studies only looked at the association between ANA and infections among ANA positive individuals (33). NHANES is designed to examine national trends in health over time and the infection assays are of high quality and comparable over time. Further, we were able to control for several important variables in examining drivers of hygiene hypothesis exposures, including age, sex, race/ethnicity, BMI, and education. In sensitivity analyses, we additionally controlled for country of birth, smoking status and household size, as these factors may influence exposure to hygiene hypothesis indicators, but the addition of these potential confounders did not meaningfully change effect estimates. Our previous work has shown that vitamin D deficiency was associated with ANA positivity in older US adults (aged 50+) (34). In the current study, however, no statistically significant differences in vitamin D levels by ANA status were observed, therefore it was not considered as a confounder. Our previous work (35) also found that some medications were associated with ANA in the general adult (18+) population, including bronchodilators in those ages 60 and older, but medication use and ANA in adolescents was not examined. A thorough examination of medication use goes beyond the scope of the current study, but is an important area of future research.

Though the results from our study were mixed, ANA may be an additional indicator of inadequate immune system education in childhood and adolescence. The immunology of the hygiene hypothesis is complex with multiple mechanisms potentially driving associations. Proper immune maturation requires antigenically rich environments; the greater frequency of self-tolerance loss in younger age groups observed in our study may be the result of reduced microbiome diversity, poor immunologic education, and molecular mimicry (29, 36). Characterizing more recent birth cohorts by ANA status, as well as allergy, atopic asthma, and infections will be an important next step in determining if trends observed in this study are maintained or diminished. In addition, as the birth cohorts examined in this study enter mid-life, monitoring for increases in autoimmune disease will shed light on the connection between early appearance of autoimmunity markers and autoimmune disease risk. Early ANA occurrence may also be a sentinel for poor immunological regulation and cellular aging is associated with ANA incidence, therefore, future studies should investigate whether children with ANA have more aged immune systems than their chronological age may represent.

## Data Availability Statement

Publicly available datasets were analyzed in this study. This data can be found here: https://www.cdc.gov/nchs/nhanes/index.htm.

## Ethics Statement

The studies involving human participants were reviewed and approved by National Center for Health Statistics (NCHS) Ethics Review Board of the Centers for Disease Control and Prevention (CDC). Written informed consent to participate in this study was provided by the participants’ legal guardian/next of kin.

## Author Contributions

Conceived of and designed the study: HM, CP, and DS. Acquired the data and performed the analysis: JW. Drafted the manuscript: HM and JW. Consulted on analytic strategy and critically revised manuscript: CP, DS, FM, and GD. All authors contributed to the article and approved the submitted version.

## Funding

This work was supported in part by the Intramural Research Program of the NIH, National Institute of Environmental Health Sciences (Z01-ES049028) and contract HHSN273201600011C to Social & Scientific Systems, and in part by the Shaw Scientist Award from the Greater Milwaukee Foundation.

## Conflict of Interest

The authors declare that the research was conducted in the absence of any commercial or financial relationships that could be construed as a potential conflict of interest.

## Publisher’s Note

All claims expressed in this article are solely those of the authors and do not necessarily represent those of their affiliated organizations, or those of the publisher, the editors and the reviewers. Any product that may be evaluated in this article, or claim that may be made by its manufacturer, is not guaranteed or endorsed by the publisher.

## Abbreviations

ANA, antinuclear antibodies; CI, confidence interval; NHANES, National Health and Nutrition Examination Survey; OR, odds ratio.
